# Salmonella Group B Ileitis Mimicking Crohn's Disease: A Case Report

**DOI:** 10.7759/cureus.52495

**Published:** 2024-01-18

**Authors:** Bereket Tewoldemedhin, Shefali Pati, Raed Atiyat, Andre Fedida, Addi Suleiman, Yazeed Abu Ruman, Ala Muhanna, Charity Iheagwara, Maria Szabela, Jihad Slim, Jack Boghossian

**Affiliations:** 1 Internal Medicine, Suburban Community Hospital (Lower Bucks Hospital), Bristol, USA; 2 Infectious Diseases, Saint Michael's Medical Center, Newark, USA; 3 Internal Medicine, Saint Michael's Medical Center, Newark, USA; 4 Gastroenterology, Saint Michael's Medical Center, Newark, USA; 5 Cardiology, Saint Michael's Medical Center, Newark, USA

**Keywords:** terminal ileitis, crohns, focal active colitis, colitis, salmonella infection

## Abstract

*Salmonellae*, considered among the enteric-fever-causing pathogens, is associated with a range of human infections, including gastroenteritis, bacteremia, and osteomyelitis. *Salmonella*-induced mesenteric adenitis and terminal ileitis resembling acute appendicitis have been reported in the literature. Here, we present a rare case of a patient presenting with severe acute active ileitis and colitis mimicking Crohn's disease with no prior history of inflammatory bowel disease and found to have *Salmonella* group B bacteremia.

## Introduction

*Salmonella* infection, which is contracted through contaminated eggs, dairy products, poultry, or ground meat, as well as contact with exotic reptile pets, is considered among the most frequent causes of foodborne illnesses in the United States [1,2]. The majority of *Salmonella* infections induce self-limited acute gastroenteritis, but severity can range from mild to severe invasive infections with regional mesenteric adenitis and terminal ileitis [2,3]. Within the complex fabric of gastrointestinal illnesses, the relationship between Crohn's disease and terminal ileitis caused by *Salmonella* presents a fascinating focus point for both healthcare providers and researchers. The appearance of terminal ileitis with crypt abscesses is an interesting parallel between *Salmonella*, a bacterial pathogen known to cause foodborne infections, and Crohn's disease, a chronic inflammatory disorder affecting the digestive system. There is a notable commonality in the clinical presentations of the two illnesses; patients report symptoms including weariness, diarrhea, stomach discomfort, and weight loss [3,4]. It has been postulated that with inflammatory bowel disease, the major pathology arises from the dysregulated immune response resulting from the interaction of environmental and genetic risk factors [4]. Infections with pathogenic bacteria are a key factor that can contribute to the development of inflammatory bowel disease [4,5]. Differentiating between an acute pathological response due to the infectious agent and the development of an inflammatory bowel disease can oftentimes be challenging based on clinical presentation and pathology findings alone, which can have prognostic and therapeutic implications [4,5].

## Case presentation

A previously healthy 19-year-old female with a remote history of hemorrhoids presented to the emergency department (ED) with complaints of bloody stools and abdominal pain for three days. The patient states that one day prior to the onset of her symptoms, she had eaten out at a sea food restaurant with friends, and about 1 hour after her meal, she started to feel periumbilical abdominal pain, quickly followed by multiple bouts of watery diarrhea, which then became bloody over the next three days. She also stated that she has low-grade fevers at home. She had tried over-the-counter antacids for the abdominal pain with no improvement, prompting the ED visit. No other family members or friends exhibited similar symptoms.

Her vital signs were as follows: temperature of 98.7, heart rate of 60 bpm, blood pressure of 111/55 mmHg, respiratory rate of 16 breaths per minute, and oxygen saturation of 98% on room air. A physical exam revealed mild epigastric tenderness, otherwise unremarkable. Laboratory results showed mild leukocytosis and mild increases in acute-phase reactants with elevated erythrocyte sedimentation rate (ESR) and C-reactive protein. The complete metabolic panel was unremarkable (Table 1).

**Table 1:** Laboratory results for the patient

Parameters	Results	Reference values
Hemoglobin	12.6 g/dl	12-16 g/dl
Platelets	429,000/µL	150,000-400,000/µL
White blood cell	14,500/µL	3100-10,500/µL
Neutrophils	8600/µL	1500-7000/µL
Neutrophils (relative percent)	64%	40%-60%
Lymphocytes	4100/µL	1000-4000/µL
Lymphocytes (relative percent)	28%	20%-40%
Monocytes	1500/µL	300-900/µL
Monocytes (relative percent)	10.5%	4%-8%
Aspartate Transaminase	32 IU/L	10-36 U/L
Alanine Transaminase	27 IU/L	10-49 U/L
Erythrocyte sedimentation rate	26 mm/hour	0 - 20 mm/ hour
C-reactive protein	1.2mg/dl	0.0 - 0.8 mg/dL
Glucose	74 mg/dl	70-140 mg/dl
Blood urea nitrogen	20 mg/dl	6.0-24 mg/dl
Creatinine	0.7 mg/dl	0.5-1.0 mg/dl
Sodium	139 mmol/L	136-145 mmol/L
Potassium	3.6 mmol/L	3.5-5.3 mmol/L
Chloride	102 mmol/L	98-110 mmol/L
Albumin	3.5 g/dl	3.6-5.1 g/dl
Lipase	25 U/L	12-53 U/L

The stool exam was negative for the ova and parasite examinations. In addition, rotavirus antigens, giardia and cryptosporidium antigens, and Clostridium difficile were also negative. 

The abdominal ultrasound performed was negative for any acute pathological changes within the abdomen. In addition, urine pregnancy tests, HIV tests, and viral hepatitis markers were all negative. 

**Figure 1:**
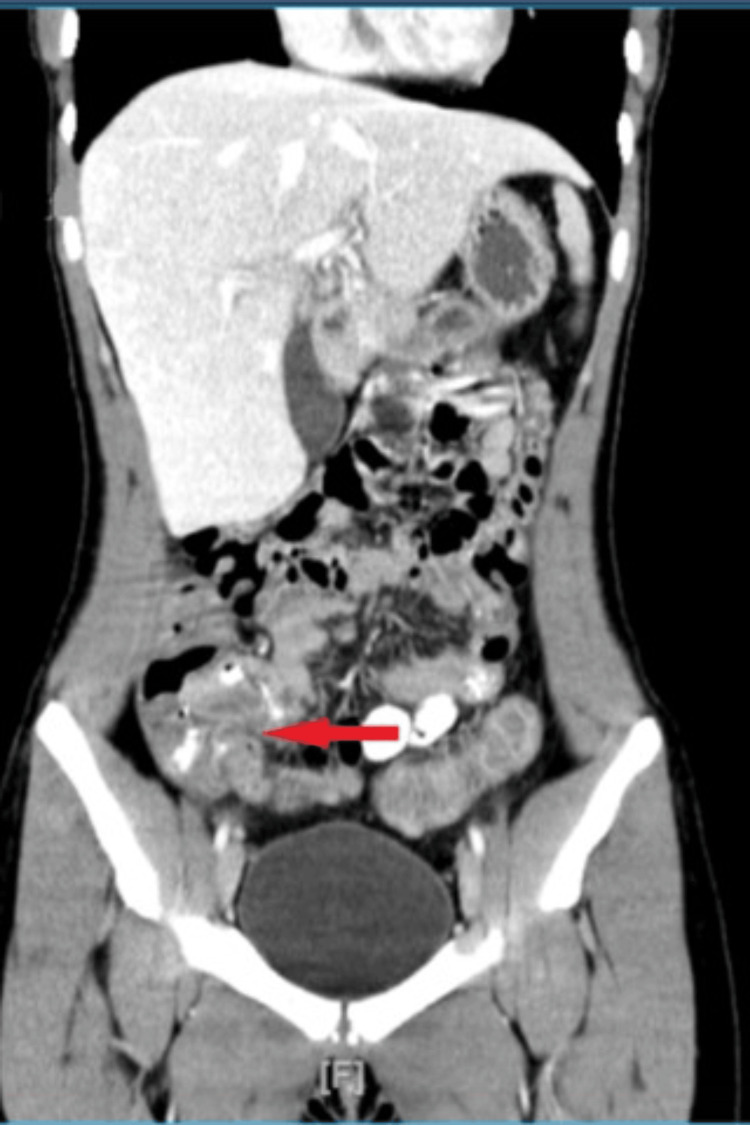
Computer tomography (CT) showing thickening of the terminal ileum (red arrow)

Computer tomography of the abdomen showed mild hepatomegaly and thickening of the terminal ileum, which was suggestive of an inflammatory process (Figure 1). 

**Figure 2:**
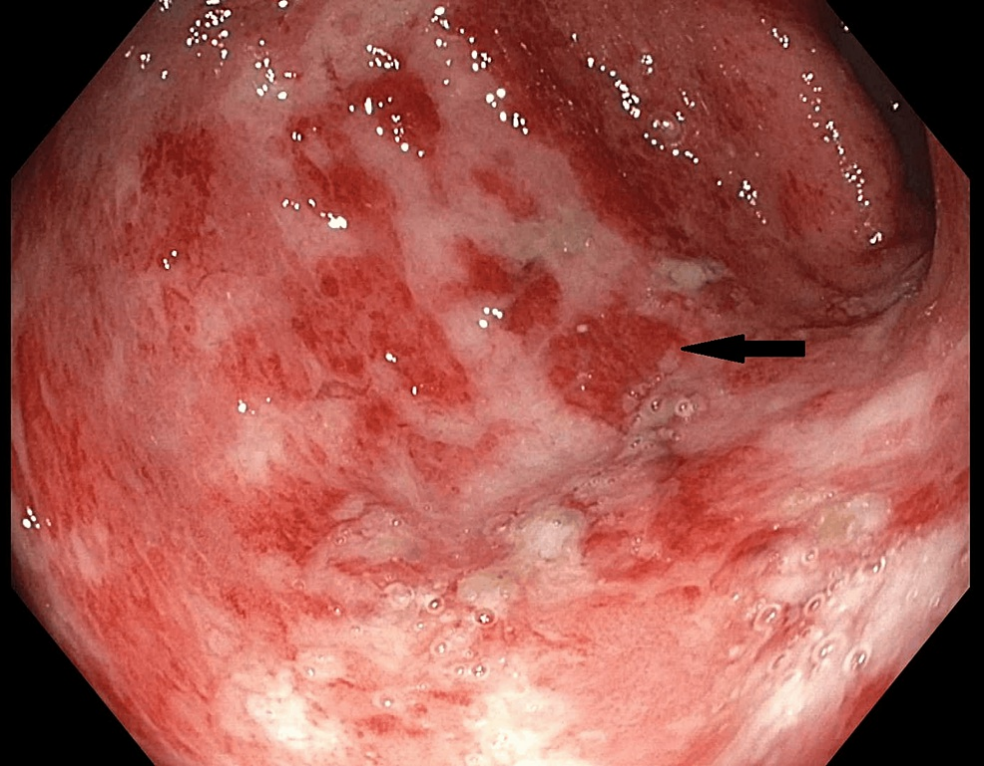
Colonoscopy image showing ulcerated mucosa of the sigmoid colon (black arrow)

A colonoscopy was subsequently performed, which showed discontinuous areas of non-bleeding ulcerated mucosa with no stigmata of recent bleeding within the sigmoid colon (Figure 2).

**Figure 3:**
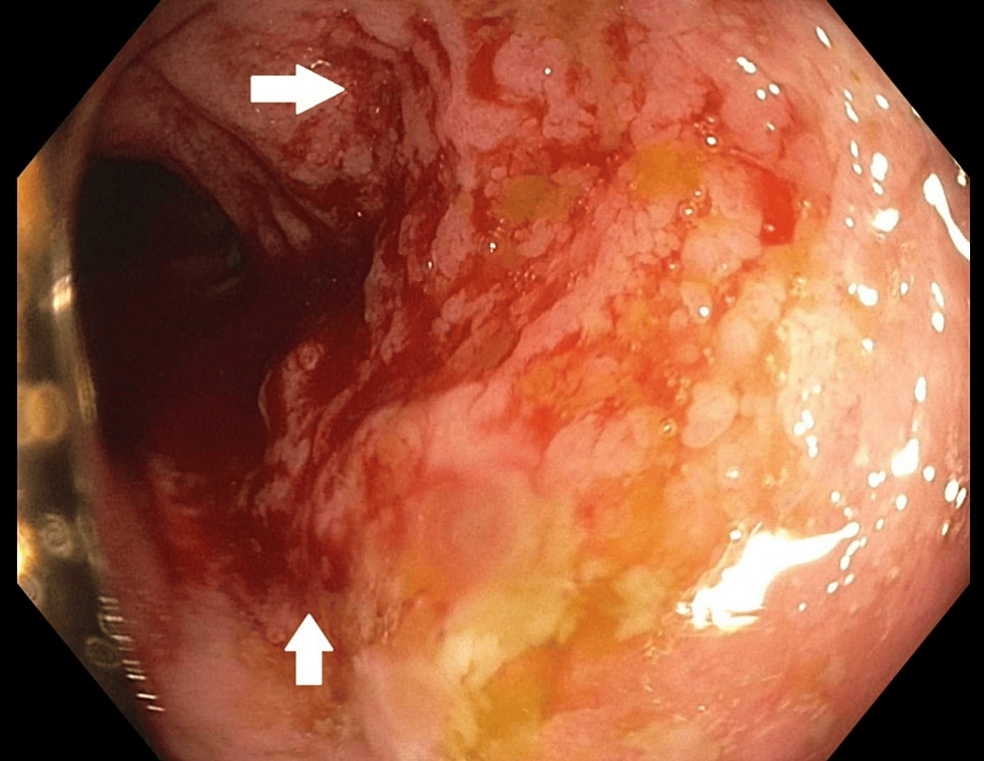
Colonoscopy images demonstrating severe ileitis of the terminal ileum (white arrows)

Patchy, severe inflammation with friable and serpentine ulcerations was found within the descending colon and transverse colon, suggesting inflammatory bowel disease. Patchy inflammation with friable serpentine ulcerations was found in the terminal ileum, suggesting severe ileitis (Figure 3).

**Figure 4:**
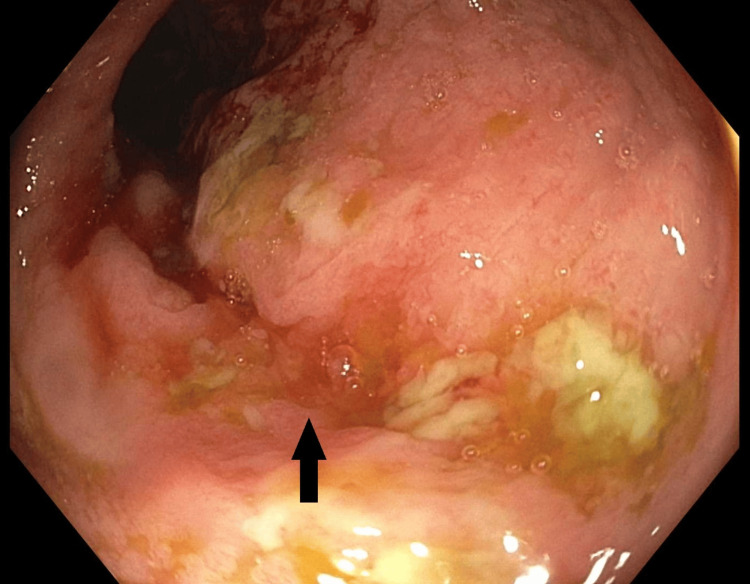
Patchy inflammation with congestion and edema of the terminal ileum (black arrow)

Patchy congestion with edema and erythema was also visible on the terminal ileum, suggesting severe underlying inflammation (Figure 4).

Biopsies were taken for histopathology from all the sites. The pathology results of the ileal biopsy showed severe chronic active ileitis with focal ulceration and lymphoid aggregates. That of the descending colon of the large intestine showed severe chronic active colitis with superficial ulcers, cryptitis with crypt abscesses, and lymphoid aggregates. The pathology results of the sigmoid colon showed severe chronic active colitis with superficial ulceration, cryptitis, and crypt abscess. With the above findings, she was initially placed on steroids, but she continued to have abdominal pain and diarrhea. 

The inflammatory disease expanded panel, which included ASCA (anti-Saccharomyces cerevisiae antibodies), ALCA (antilaminaribioside carbohydrate antibodies), ACCA (antichitobioside carbohydrate antibodies), and AMCA (antimannobioside carbohydrate antibodies), was all negative. 

At this point, the blood cultures collected from the patient yielded positive results for *Salmonella* Group B species with susceptibility to fluoroquinolones. The steroids were discontinued, and she was placed on ciprofloxacin 400mg intravenous twice a day, with significant improvement in her symptoms. She was then transitioned to ciprofloxacin 500mg oral twice a day for a 14-day course with outpatient follow-up with infectious disease and gastroenterology offices with complete resolution of her symptoms. 

## Discussion

Reports of ileitis and colitis brought on by the *Salmonella* group B serogroup are not uncommon [2-4]. Although most cases of *Salmonella* gastroenteritis are acute, self-limited, and have a brief course, severe *Salmonella* enteritis usually shows detectable neutrophilic infiltration in failing crypts, particularly in the lamina propria [5,6]. But there are also areas of ulceration and bleeding that mimic Crohn's disease. Large areas of bleeding and mucosal and submucosal necrosis, together with punched-out mucosal ulcerations or erosions with elevated margins, are specific features seen with *Salmonella colitis* [5-7]. 

A member of the inflammatory bowel disease (IBD) family, Crohn's disease is defined by persistent inflammation of the gastrointestinal system with a tendency to involve the terminal ileum [2,3]. Nonetheless, a set of bacteria known to be linked to foodborne infections, *Salmonella*, has been found to be a possible cause of both acute and occasionally chronic inflammation in the terminal ileum, leading to a state that has a striking resemblance to Crohn's disease [6-8]. Differential diagnosis in Crohn's disease is based on microbial isolation, the amount and frequency of fecal blood recurrence, and primarily on typing of the inflammatory infiltration, which in salmonellosis is neutrophilic as opposed to lymphocyte-plasmacyte and usually granulomatous infiltration in Crohn's disease [6,7]. It can become quite challenging to differentiate between the different clinical, procedural, and histologic characteristics of infectious gastroenteritis and IBD. Managing a case of undiagnosed IBD aggravated by infectious gastroenteritis may exacerbate these challenges [4,5]. *Salmonella* infections are a serious health concern and a common source of food-borne gastroenteritis outbreaks [6,7]. Further supporting colonoscopy in situations like the one above is the fact that the absence of *Salmonella* in the stool alone does not always indicate a lack of infection. The small intestine is usually thought to be the primary location affected by human salmonellosis [4,5]. But, colonic involvement is possible, particularly in cases of non-typhoid salmonellosis [5]. Diffuse colitis is typically caused by typical *Salmonella* infections with radiographic and endoscopic characteristics typically resembling ulcerative colitis [5,6]. It is rare for endoscopic results from *Salmonella* infections to indicate Crohn's disease. 

The confluence of these two separate etiologies results in a shared pathology that is defined by the development of crypt abscesses and terminal ileitis [7]. The terminal ileum acts as a focal point for immune surveillance and nutritional absorption, and trying to understand the similarities between Crohn's disease and terminal ileitis caused by *Salmonella* necessitates a detailed investigation of the immune responses elicited by these disease entities [5,6]. Both disorders cause an overreaction by the immune system, which results in mucosal injury, persistent inflammation, and the development of small pockets of infection inside the intestinal crypts known as crypt abscesses [8-10]. The pathophysiological similarities between *Salmonella*-induced terminal ileitis and Crohn's disease are noteworthy, as shown by this common histological characteristic observed [11,12]. Additionally, on imaging or endoscopy, it may be difficult to distinguish between various causes of ileitis, with biopsy being helpful in demonstrating acute ileitis [11-13]. Culture remains the foundation for a conclusive diagnosis of *Salmonella* [11,13]. 

Our patient was a young teenage woman with no significant previous medical history who was admitted due to severe acute enteritis. The severity of her symptoms, in combination with the fact that the results of stool cultures were unavailable at the time, mandated CT. An abnormal CT finding was the prompted colonoscopy, with findings consistent with inflammatory bowel disease, most in keeping with Crohn’s disease, and later findings of blood culture that were positive for *Salmonella* infection, prompting antibiotic therapy and significant improvement of her symptoms.

## Conclusions

Patients who present with features suggestive of inflammatory bowel disease and possible infectious colitis remain a diagnostic challenge, as elucidated by this case. *Salmonella* group B continues to be one of the major causes of colitis that can mimic the colonoscopy and pathology features of Crohn’s disease. Thus, if not recognized and treated early, it may result in significant morbidity with life-altering complications. Only time will tell if the antecedent *Salmonella* infection caused a reactive inflammatory bowel disease in our patient. Early recognition coupled with early therapy in these patients can significantly improve outcomes and change the course of the illness. 

## References

[1] Giannella RA. Salmonella. In: Baron S, editor editor (1996). Medical Microbiology.

[2] Kahlon Kahlon, Arundeep MBBS; Williams, Renee MD (2012). Salmonella Presenting as Ulcerative Colitis.

[3] Schultz BM, Paduro CA, Salazar GA (2017). A potential role of Salmonella infection in the onset of inflammatory bowel diseases. Front Immunol.

[4] Gradel KO, Nielsen HL, Schønheyder HC, Ejlertsen T, Kristensen B, Nielsen H (2009). Increased short- and long-term risk of inflammatory bowel disease after salmonella or campylobacter gastroenteritis. Gastroenterology.

[5] Crum-Cianflone NF (2008). Salmonellosis and the gastrointestinal tract: more than just peanut butter. Curr Gastroenterol Rep.

[6] Balthazar EJ, Charles HW, Megibow AJ (1996). Salmonella and Shigella-induced ileitis: CT findings in four patients. J Comput Assist Tomogr.

[7] Dilauro S, Crum-Cianflone NF (2010). Ileitis: when it is not Crohn's disease. Curr Gastroenterol Rep.

[8] Cosnes J, Gower-Rousseau C, Seksik P, Cortot A (2011). Epidemiology and natural history of inflammatory bowel diseases. Gastroenterology.

[9] Kaser A, Blumberg RS (2011). Autophagy, microbial sensing, endoplasmic reticulum stress, and epithelial function in inflammatory bowel disease. Gastroenterology.

[10] Deutsch A, Wasserman D, Ruchelli E, Johnson J, Broussard DL (1996). An uncommon presentation of Salmonella. Pediatr Emerg Care.

[11] McGovern VJ, Slavutin LJ (1979). Pathology of salmonella colitis. Am J Surg Pathol.

[12] Day DW, Mandal BK, Morson BC (1978). The rectal biopsy appearances in Salmonella colitis. Histopathology.

[13] Mogasale V, Ramani E, Mogasale VV, Park J (2016). What proportion of Salmonella Typhi cases are detected by blood culture? A systematic literature review. Ann Clin Microbiol Antimicrob.

